# Internal jugular vein catheterization: a comparative study of apical and paracarotid approaches

**DOI:** 10.1186/cc11751

**Published:** 2012-11-14

**Authors:** H Mangu, A Samantaray

**Affiliations:** 1 Sri Venkateswara Institute of Medical Sciences, Tirupati, India

## Introduction

Central venous catheterization (CVC) is a frequently performed procedure in ICUs for both monitoring and definitive central venous access. Although the apical approach is the most preferred technique in our practice, a modified landmark guided technique that uses only the carotid artery pulsation as a landmark (paracarotid approach) to locate the puncture site for internal jugular venous (IJV) catheterization attained a high success rate with few complications. The aim of the study was to compare two approaches used for IJV cannulation: the apical approach and the paracarotid approach. The primary endpoint was the rate of success. The secondary endpoints were the related adverse events and the difficulty factors (number of attempts).

## Methods

A prospective, randomized clinical trial in a tertiary-care university teaching hospital. After obtaining approval from the Hospital Ethics Committee, 50 adult patients admitted to our ICU with an absolute indication for CVC were randomized to undergo one of the two techniques. We compared the demographics, success rates, difficulty factors and adverse events.

## Results

The first-attempt success rate (80% vs. 60%, *P *< 0.05) and overall success rate (92% vs. 88%) was higher in the paracarotid group, with fewer attempts (1.08 ± 0.5 vs. 1.16 ± 0.6). There were no pneumothorax (0 vs. 5%), and less accidental arterial punctures (5% vs. 18%) in the paracarotid group, but the difference was not significant statistically.

## Conclusion

This study shows that the paracarotid approach of IJV cannulation is a better technique in providing a higher first-attempt success rate and has fewer complications.

